# Improved CO_2_/CH_4_ Separation Properties of Cellulose Triacetate Mixed–Matrix Membranes with CeO_2_@GO Hybrid Fillers

**DOI:** 10.3390/membranes11100777

**Published:** 2021-10-11

**Authors:** Chhabilal Regmi, Saeed Ashtiani, Zdeněk Sofer, Karel Friess

**Affiliations:** 1 Department of Physical Chemistry, University of Chemistry and Technology, Technická 5, 16628 Prague, Czech Republic; jamalias@vscht.cz; 2 Department of Inorganic Chemistry, University of Chemistry and Technology, Technická 5, 16628 Prague, Czech Republic; zdenek.sofer@vscht.cz

**Keywords:** gas separation, cellulose triacetate, CeO_2_@GO hybrid fillers, mixed-matrix membrane

## Abstract

The study of the effects associated with the compatibility of the components of the hybrid filler with polymer matrix, which ultimately decide on achieving mixed matrix membranes (MMMs) with better gas separation properties, is essential. Herein, a facile solution casting process of simple incorporating CeO_2_@GO hybrid inorganic filler material is implemented. Significant improvements in material and physico-chemical properties of the synthesized membranes were observed by SEM, XRD, TGA, and stress-strain measurements. Usage of graphene oxide (GO) with polar groups on the surface enabled forming bonds with ceria (CeO_2_) nanoparticles and CTA polymer and provided the homogeneous dispersion of the nanofillers in the hybrid MMMs. Moreover, increasing GO loading concentration enhanced both gas permeation in MMMs and CO_2_ gas uptakes. The best performance was achieved by the membrane containing 7 wt.% of GO with CO_2_ permeability of 10.14 Barrer and CO_2_/CH_4_ selectivity 50.7. This increase in selectivity is almost fifteen folds higher than the CTA-CeO_2_ membrane sample, suggesting the detrimental effect of GO for enhancing the selectivity property of the MMMs. Hence, a favorable synergistic effect of CeO_2_@GO hybrid fillers on gas separation performance is observed, propounding the efficient and feasible strategy of using hybrid fillers in the membrane for the potential biogas upgrading process.

## 1. Introduction

Membrane systems have become an accepted gas treating technology. This technology plays a key role in biogas upgrading and offshore natural gas treatment processes, especially to remove CO_2_ [1]. Depending on the materials used for the synthesis, inorganic and polymeric membranes are in use. Inorganic membranes show significantly higher diffusivity and selectivity of gas molecules due to discerning ability based on pore size and shape, possessing high thermal and chemical stability, mechanical strength, and longer life span [2]. However, these membranes face the limitation of poor scalability, high cost and complicated fabrication procedure [3].

On the other hand, polymer membranes still dominate the current market in the separation process. Despite this, pristine polymeric membranes always suffer from the trade-off between permeability and selectivity [4]. The high demand for membrane technology in gas separation and rapid effort toward seeking membranes with higher permeability and selectivity has motivated the development of MMMs. The addition of the porous/nonporous inorganic fillers into the polymer matrixes combine the processability of polymeric membranes with the good gas separation performance of the inorganic membranes [5], synergistically contributing to the enhancement in membrane separation performance, thus minimizing the trade-off limit. Furthermore, highly absorptive inorganic nano-fillers benefits from their large surface area and abundant active sites [6]. However, these inorganic fillers occasionally exhibited limited compatibility with the polymer matrixes, poor dispensability due to their strong intermolecular van der Waals interaction and aggregation in membranes [7,8]. The resulting difficulty in achieving homogeneous fillers dispersion in a continuous polymer phase and consequent loss of selectivity thus represent the major limitation of MMMs preparation. Furthermore, the functionalization of fillers is often conducted to improve their compatibility and dispersion properties in the polymer matrix [9]. However, this technique is laborious and could disrupt the structure and lose the intrinsic properties of the native fillers particles [10]. Much research into potential new materials for gas separation membranes is thus driven by the limitation and disadvantages which is in existence in the current available MMMs.

Coronas group reported a significant improvement in the CO_2_/CH_4_ and CO_2_/N_2_ selectivity with hybrid MOF (HKUST-1)@zeolite (silicate-1) and polysulfones system [11]. They also conducted experiments to investigate the synergistic effect of two fillers with different natures [11,12,13]. Since then, the use of the two different filler particles into a polymer matrix to enhance the gas separation performance of the resultant MMMs has attracted much attention. Jamil et al. fabricated the MMMs by incorporating reduced graphene oxide (rGO) and ZIF-8 nanofillers into the PES matrix and observed high permeability and low selectivity for CO_2_/CH_4_ [14]. Wong et al. synthesized the highly dispersed CNT into a thin-film nanocomposite via the addition of an amphiphilic GO nanosheet onto a PSF support layer. They found 30% and 60% improvement in the gas permeability and selectivity, respectively [15]. Ahmed et al. prepared the PVDF membrane incorporated with POSS and SAPO-34 zeolite and observed the detrimental increment in CO_2_ flux compared to the pristine PVDF membrane [16]. It was revealed that the interactions between the two different fillers with distinct properties, morphologies, and surface chemistry promote polymer-fillers interaction, thereby reducing the probability of the fillers agglomeration and enhancing the gas separation efficiency leading to synergy to acquire non-linear effects [13]. Nevertheless, only a countable number of such studies can be found in the literature, suggesting that such hybrid filler MMMs are still in the initial stage of development, and further research is essential. 

In this study, we investigate the synergy between CeO_2_ and GO nanosheets in MMMs. GO was selected as a filler because the sheeted morphology of GO can improve the dispersion of the fillers co-existed in the nanocomposite. Such behavior is assumed due to the structural and property-wise resemblance of GO as polyelectrolytes in a 2D configuration. Their surfactant-like characteristics at the interface thus can serve as a special class of dispersion agents [17]. Likewise, these nanosheets act as a potential support to deposit inorganic oxides nanoparticles on their surface due to the presence of a large number of polar groups that play an important role in participating in a wide range of bonding interactions [18]. Similarly, the charge transferability between GO and CeO_2_ due to the difference of their work function create stronger interaction between them [19]. Due to the diverse reactive groups like epoxide, hydroxyl, and carboxylic acid at the edge and basal plane of GO nanosheets, there is high compatibility among the GO nanosheets with the polymer matrix via covalent or non-covalent connections [20]. Moreover, they tend to be parallel to the membrane surface. Such sheeted GO morphology gave rise to a strong steric effect and prevented the aggregation of other fillers [21]. Furthermore, the randomly distributed GO sheet may act as a barrier in the polymer matrix due to the hindered diffusion pathway through the nanocomposite, thus enhancing the selectivity [14,15]. GO, which retains the lamellar structure of graphite, contains several unpaired π electrons and oxygenated functional groups, making GO highly selective towards small and polar molecules like CO_2_ [22]. On the other hand, Cerium oxide (CeO_2_), one of the most reactive rare earth metal oxides, has received attention as a promotor or catalyst even in industrial processes due to its oxygen storage capacity [23]. Similarly, owing to the high adsorption affinity of both oxidized/reduced forms of CeO_2_ with CO_2_ forming bridged, monodentate, bidentate carbonates, and bicarbonates, and leading to the improved permeability when blended with polymer matrix [24,25], CeO_2_ was selected as another material for the formation of composite nanofillers. The synthesized membrane’s affinity was then determined by evaluating the CO_2_ uptake and CO_2_ and CH_4_ permeability capacity. To the best of our knowledge, there is no evidence of using CeO_2_@GO hybrid filler to fabricate MMMs in conjunction with polymer for gas separation purposes yet.

## 2. Materials and Methods

### 2.1. Chemicals

CTA (acetyl content 43–44%) was obtained from Acros Organics (Waltham, MA, USA). Cerium nitrate hexahydrated (Ce(NO_3_)_3_·6H_2_O, 99.99%), sodium hydroxide (NaOH, 98.99%), and 1-methyl-2-pyrrolidinone (NMP, ACS reagent > 99.0%) were purchased from Sigma-Aldrich (St. Louis, MO, USA). Ethanol (C_2_H_5_OH, 99.89%) was purchased from VWR Chemicals (Radnor, PA, USA). All the chemicals were of analytical grade and were used as received without any further purification. Similarly, GO was synthesized in the laboratory. The detail of the synthesis process is explained in supplementry information section.

### 2.2. Preparation of CeO_2_@GO Hybrid Fillers

CeO_2_ nanoparticles were prepared using the facile hydrothermal process [25]. 0.1 g of as-prepared CeO_2_ nanoparticles were mixed with 130 mL ethanol, and different dopamine functionalized GO concentrations (3 wt.%, 5 wt.%, 7 wt.%, and 10 wt.% with respect to the wt. of CeO_2_ nanoparticles). The mixture was sonicated for 30 min. (VWR ® Ultrasonic cleaner, USC-THD (Lutterworth, UK) stirred for 4 h. followed by hydrothermal treatment using Teflon lined vessel at 110 °C for 18 hrs. Afterwards, the mixture was dried at 80 °C in an oven.

### 2.3. Preparation of CTA-CeO_2_@GO Mixed-Matrix Membrane and Its Mechanism of Formation

0.045 g of the hybrid nanoparticles (CeO_2_@GO) was mixed with 26 mL of NMP. The mixture was sonicated for 30 min. and stirred for 3 h afterwards. 1.62 g of CTA polymer was added to the initial mixture and stirred for 18 h to obtain the optimal dispersion of nanoparticles in the polymer solution. The mixture was further sonicated for 30 min. and left undisturbed for 4 h. The membrane was then cast in a glass plate using an applicator (Elcometer 3580, Manchester, UK). The casted film was left undisturbed at ambient conditions till complete evaporation of the solvent took place. The membrane was then further kept in a vacuum oven at 60 °C for overnight for complete drying. The membrane with 3 wt.%, 5 wt.%, 7 wt.%, and 10 wt.% of GO are referred to as CTCeGO3, CTCeGO5, CTCeGO7, and CTCeGO10, respectively, throughout the manuscript. The physico-chemical properties and the gas sorption/ permeation behavior of CTA-CeO_2_ and pristine CTA membranes were reported previously [25]. 

Among various techniques of membrane synthesis, the dry-casting method was used. This technique is a suitable method for preparing asymmetric membranes with dense skin applicable for the gas separation process. This synthesis process is characterized by nonsolvent and /or solvent evaporation from an initially homogeneous single-phase polymer solution. During solidification, polymer from the polymer-rich phase precipitates to form a solid matrix which enfolds the polymer–lean phase. The final membrane thickness is a fraction of the initial cast film thickness owing to both nonsolvent and/or solvent loss and excess volume of mixing effects [26]. Similarly, membrane morphology and performance are also strongly influenced by external conditions such as the casting and evaporation temperature and air circulation during the evaporation step. Since NMP is a very slow evaporating solvent, the composition change in the evaporation process is rather slow. The evaporation of NMP from the surface of the casting solution can be assumed to be compensated by NMP diffusion from the casting solution interior to the surface. This slow solvent evaporation at ambient temperature and ambient airflow results in the membranes with the thin dense skin layer desirable for gas separation [27]. 

### 2.4. Materials Characterization

The transmission electron microscopy (TEM) analysis of the nanoparticles was performed on a JEM-2200FS (Jeol, Kyoto, Japan) instrument, maintaining an accelerating voltage of 200.00 kV in TEM imaging mode. Similarly, the morphology of prepared membranes was determined using scanning electron microscopy (SEM, Tescan LYRA, Brno, Czech Republic, 15 kV accelerating voltage, SE detector) equipped with energy dispersive spectroscopy (EDS, Oxford Aztec, 80 mm^2^, High Wycombe, Abingdon, UK) for the detailed analysis of element distributions within the materials and chemical microanalysis of elements present. The 3D non-contact optical surface profiler New View 9000 (ZYGO, Middlefield, CT, USA) was used for the no-contact surface roughness measurement. X-ray photoelectron spectroscopy (XPS) analysis was carried out in ESCAProbeP Spectrometer, Omicron Nanotechnology (Uppsala, Sweden). The primary X-ray beam was monochrome radiation of an Al lamp with energy of 1486.7 eV. Powdered X-ray diffraction (XRD) measurement was performed at a temperature of 273.5 K using a 2nd-Generation D2 Phaser X-ray diffractometer (Bruker, Billerica, MA, USA) with Cu Kα radiation (λ = 0.15418 nm), SSD (1D mode) detector, coupled 2θ/θ scan type, and continuous PSD fast scan mode. The range of measured Bragg angles was 5°–80°. Fourier-transform infrared spectroscopy (FTIR) measurements were performed using a NicoletTM iS50 FTIR Spectrometer (Thermo Fischer Scientific, Waltham, MA, USA) in absorbance mode. The spectra were taken in the wavenumber range of 400–4000 cm^−1^. The thermogravimetric analysis (TGA) of synthesised MMMs was done by Setsys Evolution (Setaram Lyon, France). The samples were heated in an aluminium crucible from 40 °C to 800 °C at a rate of 10 °C·min^−1^ under an N_2_ atmosphere with a flow rate of 60 mL·min^−1^. Tensile stress-strain curves were measured using an Instron Universal Testing Machine 3365 (Instron, Norwood, MA, USA) equipped with pneumatic grips, rubber-coated sample gauge length (initial sample length between the grips) of 10 mm, sample width of 4 mm (on average), and a crosshead speed of 5 mm·min^−1^ until specimen break. The measurement was carried out at an ambient temperature. 

### 2.5. Gas Sorption and Gas Permeation Measurements

CO_2_ and CH_4_ sorption experiments were performed gravimetrically at 25 °C in a pressure range from 0.1 to 1.5 MPa using a self-developed sorption apparatus equipped with a calibrated (McBrain) quartz spiral balance. A detailed description of the apparatus and the experimental procedure is described elsewhere [28,29]. The gas permeation affinity of all synthesized membranes was determined by using single gases (H_2_, CO_2_, O_2_, N_2_, and CH_4_) in a custom-built time lag permeation setup. The membrane for gas permeation measurement was cut and tightly enclosed in a circular membrane permeation cell with an effective membrane surface area of about 2.14 × 10^−4^ m^2^. Similarly, the permeation cell was made air-free by using a vacuum at both ends. In addition, after each gas experiment, the permeation cell was continuously evacuated with a vacuum to remove the gases present therein. The synthesized membranes were subjected to test gases, and the data were collected using the SWeTr version 1.13 (2003, Neovision, Prague, Czech Republic) data acquisition software. All permeation data were collected when the steady-state was reached. Each gas was measured three times, and the average value was recorded to minimize the experimental error [25]. 

The increase in the pressure in the fixed permeate volume was monitored as a function of time as soon as the membrane was exposed to feed gas at a pressure of 1.5 bar. For the given setup, the transient permeation curve, describing the pressure increase on the permeate side after exposure of the membrane to the feed gas, takes the following form [30,31].

(1) $$P_{t}=P_{0}+{\left(\frac{d_{p}}{d_{t}}\right)}_{0}t+\frac{RTAl}{V_{p}\;V_{m}}p_{f\;}S\left(\frac{D_{t}}{t^{2}\;}-\frac{1}{6}-\frac{2}{\pi^{2}\;}\sum_{1}^{\infty }\frac{{\left(-1\right)}^{n}}{n^{2}}\;exp\left(-\frac{Dn^{2\;}\pi^{2\;}t\;}{t^{2}}\right)\right)$$

where *P_t_* is the permeate pressure (bar) at time *t*(s); *P*_0_ is the initial pressure, which is usually less than 0.05 mbar; *(d_p_/d_t_)*_0_ is the baseline slope, which is normally negligible for a defect-free membrane; *R* is the universal gas constant (8.314 × 10^−5^ m^3^ bar mol^−1^ K^−1^); *T* is the absolute temperature (K); *A* is the active membrane area (m^2^); *V_p_* is the permeate volume (m^3^); *V_m_* is the molar volume of a gas at standard temperature and pressure (22.4 × 10^−3^ m^3^ STP mol^−1^ at 0 °C and 1 atm); *p_f_* is the feed pressure (bar); *S* is the gas solubility (m^3^ STP m^−3^ bar^−1^); *D* is the diffusion coefficient, and *l* is the membrane thickness.

In the steady-state permeation condition, the exponential term approaches zero; hence, the equation becomes:

(2) $$P_{t}=P_{0}+{\left(\frac{d_{p}}{d_{t}}\right)}_{0}t+\frac{RTA}{V_{p}V_{m}}\times \frac{p_{f}P}{l}\left(t-\frac{l^{2}}{6D}\right)$$

A plot of *P_t_* versus *t*, after a long time, produces a straight line, which, upon extrapolation, intersects the time axis at $t=\frac{l^{2}}{6D}$, which describes the time lag (Ѳ) in permeation. These equations thus allow for the calculation of diffusion and permeability coefficients. Assuming the validity of the solution-diffusion model, the solubility coefficient was then determined indirectly by a simple relation; P=S×D.

The gas permeability (*P*, 1 Barrer = 10^−10^ cm^3^ (STP) cm·cm^−2^·s^−1^·cmHg^−1^) is expressed by the following equation:

(3) $$P=\frac{lQ_{i}}{A\Delta P_{i}}$$

where *l* refers to the thickness of the membrane (µm), *Q* represents the volume flow rate (cm^3^·s^−1^, STP) of gas *i*, *A* is the effective membrane area (cm^2^), and *ΔPi* is the partial pressure difference across the membrane (cmHg). Similarly, the selectivity is expressed as:

(4) $$\alpha_{ij}=\frac{P_{i}}{P_{j}}$$

where $P_{i}\;$ and $P_{j}$ are the permeability of two pure gases ($P_{i}\;$> $P_{j}$), respectively.

## 3. Results

### 3.1. Physico-Chemical Characterizations

**Figure 1:**
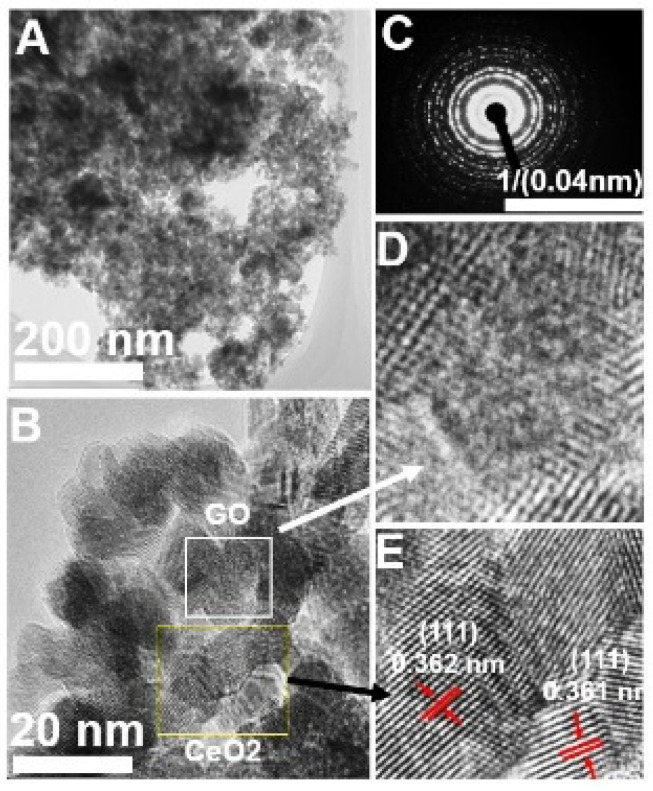
(**A**) TEM images of CeO_2_@GO composite; (**B**) HRTEM image shows both CeO_2_ and GO region; (**C**) SAED of the CeO_2_; (**D**,**E**) Enlarged HRTEM images displaying the corresponding GO and CeO_2_ lattice structure in the selected area in B.

Structural characterization using TEM provided insights into CeO_2_@GO composite filler. Figure 1 shows the typical TEM images of the CeO_2_@GO composite filler. Figure 1A displays a low magnification TEM image, where GO nanoparticles show a darker colour contrast on the CeO_2_ surface. Figure 1B exhibit a high-resolution TEM (HRTEM) image. It can be seen that the CeO_2_ and GO particles were uniformly dispersed one into another. The distinct lattice spacing of 0.362 ± 0.001 nm in the enlarged section (Figure 1E) corresponds to the (111) plane of CeO_2,_ indicates the formation of the CeO_2_@GO matrix. The SAED pattern (Figure 1C) were assigned to the reflection of the CeO_2_ and (002) of GO which agrees well with the XRD pattern. The EDS results (Figure S1) demonstrate the presence of Ce, O and C on the composite matrix. The corresponding elemental mapping confirms the homogeneous distribution of elements Ce, O and C on the matrix. 

**Figure 2:**
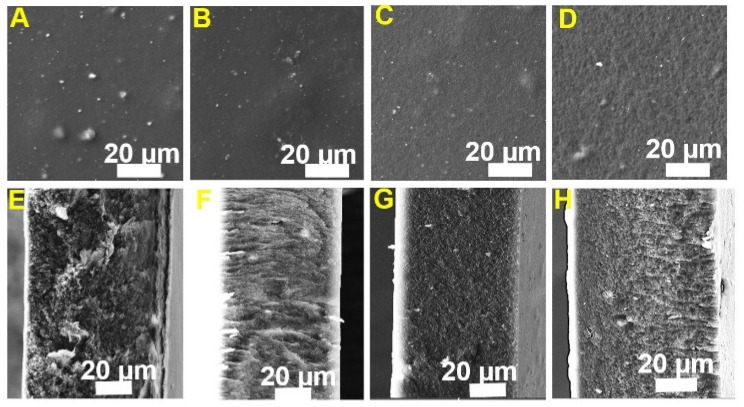
SEM images of the surface and their corresponding cross-section of the synthesized samples; (**A**,**E**) CTCeGO3, (**B**,**F**) CTCeGO5, (**C**,**G**) CTCeGO7 & (**D**,**H**) CTCeGO10 respectively.

SEM images of the surface and cross-section of synthesized MMMs are shown in Figure 2. The bright spots in the images represent the embedded CeO_2_@GO nanoparticles. As can be seen, CeO_2_ nanoparticles are distributed over the membrane surface with good dispersion. The tendency of CeO_2_ particles to aggregate seems to decrease with increasing GO concentration. The cross-section view showed a dense structure with few voids. The thick membranes (25 ± 10 µm) was obtained due to delayed mixing in the phase separation with the solvent evaporation [25]. Figure S2 and Table S1 depict the surface roughness analysis results of the synthesized membranes. The hybrid membrane exhibit a smoother surface with the increase in GO concentration in the blends. This decrease in surface roughness is likely attributed to the reduction in the aggregation tendency of the nanoparticles. Similarly, with the increase in the GO concentration, the membrane structure also becomes smoother. It suggests the homogeneous dispersion ability of the GO with a good polymer filler contact preventing the aggregation of the CeO_2_ nanoparticles. Furthermore, it also indicated that CeO_2_ particles were dispersed among GO nanosheets and loosely entangled in the polymer matrix. The EDS mapping of Ce over the cross-section of the CTCeGO7 sample in Figure S3 shows the homogeneous distribution of CeO_2_ throughout the membrane cross-section. The overlaid EDS image further confirms the homogeneous distribution of the fillers throughout the membrane volume, suggesting a contribution of GO to overcome the aggregation tendency of the inorganic fillers in the membrane matrix. 

**Figure 3:**
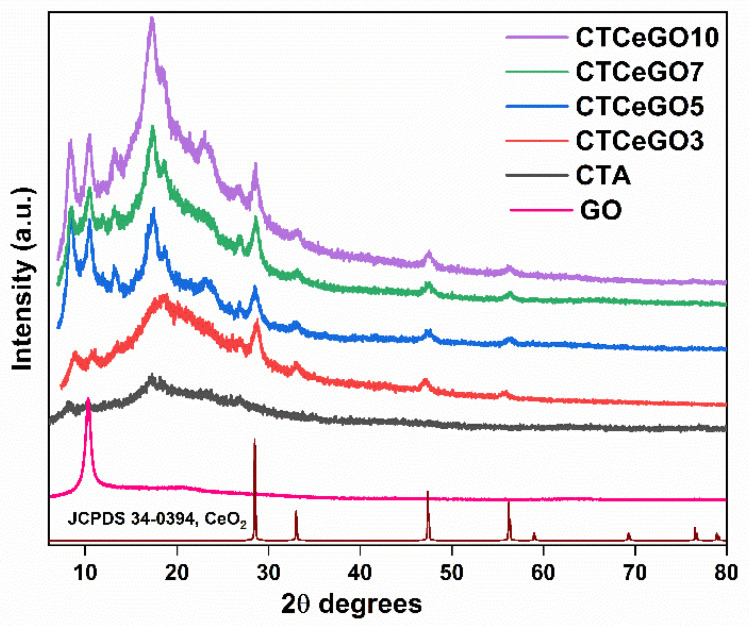
XRD patterns of the synthesized MMMs with different concentrations of GO.

The XRD patterns of pristine GO, pristine CTA, and the CeO_2_@GO incorporated CTA MMMs are displayed in Figure 3. XRD pattern of GO showed a major peak at 2θ = 10.5° that corresponds to (002) plane while for pristine CTA, a broad peak centered at 19° 2θ value is seen. For MMMs samples, all the peaks related to CTA, CeO_2_ and GO compared with pristine CTA, pristine GO, and CeO_2_ JCPDS 34-0394, respectively. This suggests the homogeneous intermixing of the hybrid filler with the polymer matrix without losing its properties and conformation.

**Figure 4:**
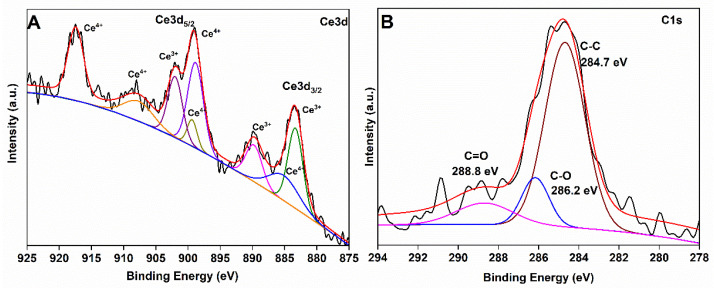
High-resolution XPS spectra of; (**A**) Ce3d and (**B**) C1s in CeO_2_@GO composite.

The chemical composition of the CeO_2_@GO composites was evaluated by XPS analysis. The overall XPS survey spectrum in Figure S4 confirmed the presence of C1s, O1s, and Ce3d elements in the CeO_2_@GO composite. The high-resolution spectrum of Ce3d_5/2_ and Ce3d_3/2_ levels in the binding energy range between 875–894 eV and 895–925 eV were resolved into eight peaks after deconvolution (Figure 4A). These peaks confirm the presence of trivalent and tetravalent oxidation states of Ce on the given composite. The presence of Ce^3+^ ions introduces oxygen vacancies in the crystal. Such high oxygen vacancies show the charge transfer effect from CeO_2_ to GO implying the covalent interaction between CeO_2_ and GO [32,33]. The C1s spectra for GO in CeO_2_@GO hybrids with characteristic peaks at 284.7 eV, 286.3 eV and 288.8 eV are attributed to the C-C, C-O and C=O groups, respectively (Figure 4B). The binding energy at 284.7 eV is a typical peak position of graphitic carbon and demonstrates the sp^2^- hybridized carbon in the graphitic state [34].

**Figure 5:**
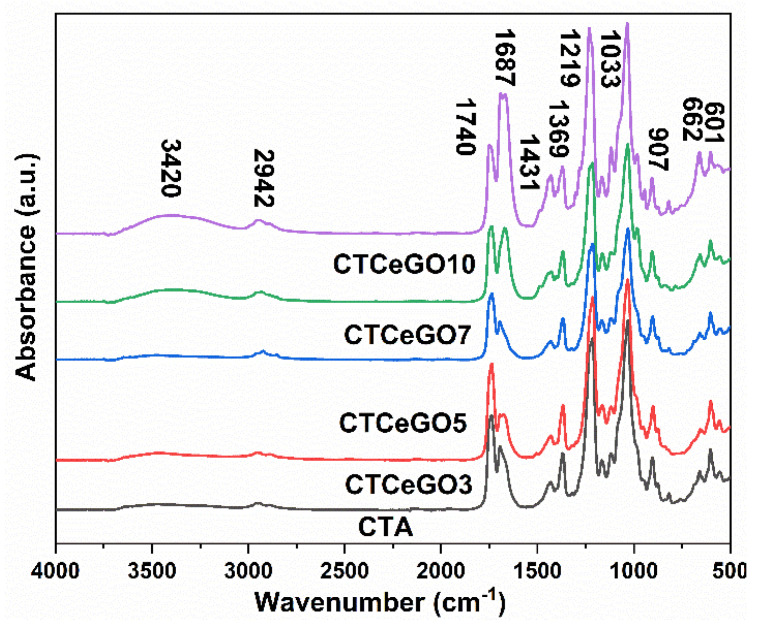
FTIR spectra of the synthesized membrane with different concentrations of GO.

Figure 5 represents the FTIR spectra of the CeO_2_@GO-CTA MMMs. The characteristic peaks at 3420 cm^−1^ refer to the O-H stretching vibration band. The peak at around 2942 cm^−1^ corresponds to the C-H bond in methyl and methylene groups. The band at 1740 cm^−1^ is associated with the stretching vibration of the C=O group, whereas the band at 1687 cm^−1^ can be attributed to the COO^−^ group. The peak at 1431 cm^−1^ is associated with the asymmetric deformation in the plane of a methyl group. The band at 1369 cm^−1^ corresponds to the C-H bending vibration of CH_3_ in the acetyl group. The absorption peaks at 1219 cm^−1^ and 1033 cm^−1^ are related to the C-O-C asymmetric and symmetric stretching band of CTA, respectively. The band at 907 cm^−1^ is attributed to C-O-C stretching at the β1-4 glycosidic linkage [25,35,36]. The spectra of pristine CTA, as well as the modified membranes, show no significant differences. Regarding the FTIR spectra of pristine GO (Figure S5), the change in intensity of peaks at 1000–1750 cm^−1^ in MMMs can be attributed to different concentrations of GO in the membrane samples.

**Figure 6:**
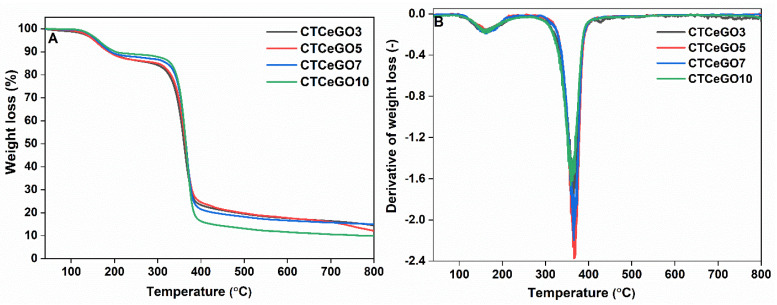
(**A**) TGA, and (**B**) its corresponding DTG plot of the synthesized MMMs.

TGA was carried out to study the influence of the hybrid nanofillers addition on the thermal stability of the MMMs. The resulting TGA and its corresponding DTA curves are presented in Figure 6. Two-step weight loss can be observed. The major weight loss in the first step occurs at around 165 °C, which is assumed due to loss of moisture, evaporation of volatile impurities within the membrane pores, and shift in the crystallinity of the glass transition temperature of the CTA matrix. The loss was around 9–14%. A significant weight loss of 85–90% was noticed at 365 °C as a result of the decomposition of the polymer matrix [37]. No significant difference in weight loss was observed for different concentrations of CeO_2_@GO loading, suggesting no detrimental effect on the overall thermal stability of the MMMs with the incorporation of the synthesized nanofillers in the low loading percentage. However, a noticeable improvement in the thermal stability effect is seen compared to the pristine CTA membrane [25].

**Figure 7:**
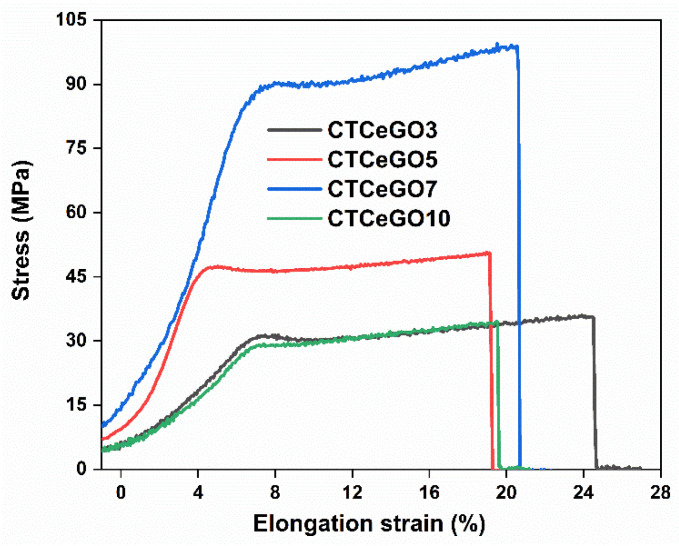
Strain versus strain graph of the CTA-CeO_2_@GO MMMs with different concentrations of GO.

Representative strain-stress curves of the synthesized MMMs showing the loading effect is depicted in Figure 7. The stress-strain curves of each sample were chosen from their corresponding data sets that are closest to their respective averages reported in Table 1. Young’s modulus increases with an increase in GO concentration, indicating the significant effect of GO on mechanical strength. The tensile strength, which characterizes sample stiffness, increases with an increase in the concentration of GO in the matrix up to 7 wt.% then after decrease at higher concentration of GO whereas elongation at maximum stress decreases. Agglomeration in the high loading of GO can be the reason for the reduction in tensile strength. Compared to pristine CTA [25], the elongation at break decreased with the addition of the hybrid fillers attributed to the rigidification of the polymer chain mainly due to favourable interaction between the polymer matrix and the nanofillers [38]. The decrease in elongation at break and increase in Young’s modulus with the increase in GO concentration manifests the role of GO in fortifying interfacial interaction and improving interfacial quality of CeO_2_@GO hybrid fillers containing MMMs.

### 3.2. Gas Separation Performances Evaluation

**Figure 8:**
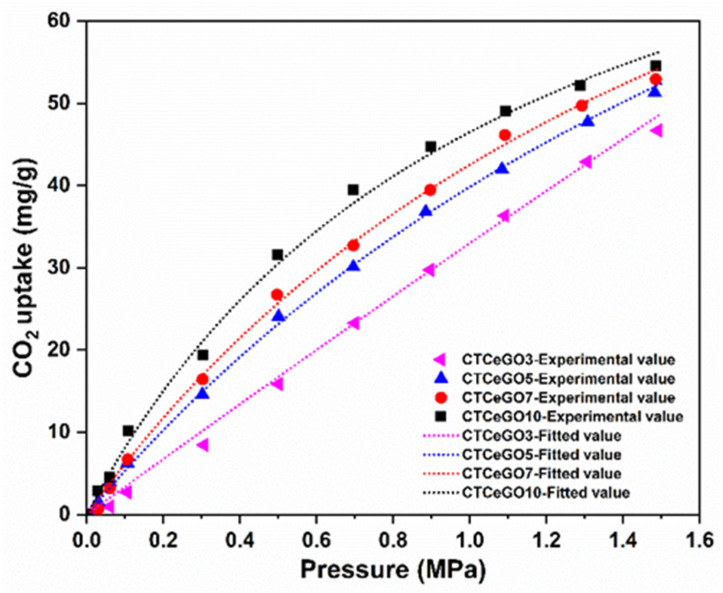
CO_2_ sorption affinity of the synthesized CTA-CeO_2_@GO membranes with different concentrations of GO.

In order to explain the elevated selectivity, high-pressure adsorption (0–1.5 MPa) tests were conducted on MMMs possessing different GO concentrations. CO_2_ uptakes for all the MMMs considerably improved with increasing pressure because of the nanofillers’ specific chemical affinity for quadrupolar CO_2_ molecules. Similarly, an increase in the concentration of the GO increases the CO_2_ adsorption amount (Figure 8). This can be attributed to the rise in the number of polar groups on the GO surface, which can uptake more CO_2_. Moreover, the CH_4_ uptake was very low, which was below the sensitivity of the measuring system. This observation is related to the high condensability of CO_2_ relative to CH_4_ and potentially to the specific interaction of CO_2_ molecules with the membrane matrix [25]. Furthermore, the sorption data were fitted to the Langmuir model [39];

(5) $$q_{e}=\frac{C_{e}K_{L}q_{m}}{1+C_{e}K_{L}}$$

where $q_{e}$(mg·g^−1^) is the equilibrium adsorption capacity, $C_{e}$ (mg·L^−1^) is the equilibrium adsorbate concentration, $K_{L}$ is the Langmuir constant (L·mg^−1^), and $q_{m}$(mg·g^−1^) corresponds to the maximum adsorption capacity of the adsorbent and gives the amount of adsorbent adsorbed after forming a complete monolayer (mg·g^−1^). The fitting parameters are enlisted in Table S2. The Langmuir model presented the best fit (R^2^ = 0.997) to describe the equilibrium data of adsorption on the given MMMs samples suggesting that the adsorption capacity on the hybrid matrix membrane occurs at energetically uniform adsorption sites with the formation of a monolayer of CO_2_ on the adsorbent surface. 

**Figure 9:**
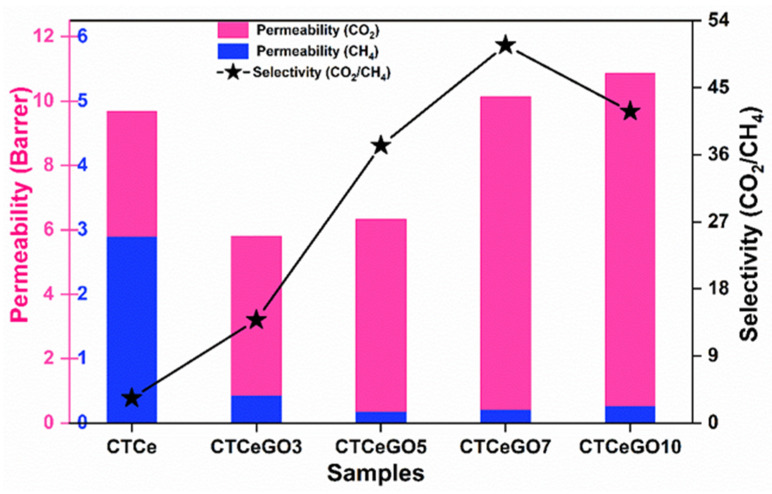
CO_2_ and CH_4_ single gas permeability and their corresponding selectivity (CO_2_/CH_4_) of synthesized MMMs.

In order to evaluate the performance of the prepared hybrid MMMs, single gas (CO_2_ and CH_4_) permeation tests were performed under the constant pressure of 1.5 bar and temperature of 25 °C. Adding a small amount of the GO into CeO_2_ to form hybrid fillers incorporating into the CTA matrix significantly increases the selectivity maintaining almost similar or slightly higher permeability than CTA-CeO_2_ MMMs (Figure 9). The gas permeation results revealed that the hybrid nanofillers significantly affect the gas separation performance of the resultant MMMs. The incorporation of hybrid CeO_2_@GO into the CTA polymer matrix causes the CO_2_ permeability and CO_2_/CH_4_ selectivity to be improved due to the high CO_2_ sorption potential and strong interfacial interaction between the fillers and polymer chains. CO_2_/CH_4_ ideal selectivity was enhanced from 3.35 to 50.7 by increasing the GO concentration up to 7 wt.%. Similarly, the gas separation efficiency of the synthesized membranes was compared with the single CTA-GO based membrane. The CO_2_ and CH_4_ permeabilities of the CTA-GO membrane was 11.29 Barrer and 0.33 Barrer, respectively, with CO_2_/CH_4_ selectivity of 34.22. A notable increment in the gas selectivity (almost 1.5 times) can be observed in the hybrid fillers based MMMs (CTCeGO7) compared to the CTA-GO membrane. The results proved that hybrid fillers caused a synergistic effect enhancing the gas separation performance compared to the single filler. Furthermore, comparing the permeability affinity of different gases H_2_, O_2_, CO_2_, N_2_ and CH_4_ were performed, taking CTCeGO7 membrane as a reference (Figure S6). The permeability follows the order as; CO_2_ > H_2_ > O_2_ > CH_4_ > N_2_. However, the kinetic diameter of CO_2_ (3.30 Å) is higher than that of H_2_ (2.89 Å) but shows higher permeability affinity, probably because of the interaction affinity with the polymer and the fillers matrix. Similarly, the selectivity of different gases pairs observed are as follows; CO_2_/N_2_ > CO_2_/CH_4_ > O_2_/N_2_ > CO_2_/H_2_. The higher selectivity of CO_2_/N_2_ can be attributed to more condensable properties and more interaction of CO_2_ molecules with the membrane matrix compared to N_2_ molecules. These results thus suggested the CeO_2_@GO hybrid fillers based MMMs to have a potential affinity for biogas/natural gas upgrading.

Due to the difference in physical properties and density between the nonporous fillers (like silica, TiO_2_, CeO_2_) and polymer matrix, the aggregation of fillers are frequently encountered. It can disrupt the polymer chain packing and increase the membrane fractional free volume, increasing gas permeability [40]. For the CTA-CeO_2_ membrane, it can thus be presumed that an increase in diffusivity and permeability (Table 2) is associated with the substantial change in the free volume due to disruption of the chain packing provides a more specious pathway for gas transport. This increase in free volume is also advantageous for large gas molecules like CH_4_, favouring increased permeability, ultimately decreasing selectivity. GO nanosheets with good dispersion properties can be easily homogeneously dispersed in a polymer matrix with good polymer fillers contact [41]. The addition of such GO sheeted morphology is presumed to give rise to a strong steric effect and prevented the aggregation of CeO_2_ fillers. These nanosheets also acted as a selective barrier to render high selectivity through the hydroxyl and carboxyl groups on the GO surface of MMMs. The interactions between the CO_2_ molecules and the polar hydroxyl and carboxyl groups on the GO surface play an important role in facilitating the transport of the CO_2_ molecules in MMMs as compared to CH_4_. Similarly, the high affinity of CeO_2_ towards CO_2_ upsurges the solubility coefficient of CO_2,_ thus resulting in an overall increase in selectivity.

Compared with CTA-CeO_2,_ the hybrid fillers incorporated membranes show a lower CO_2_ diffusivity coefficient resulting from the stronger interfacial interaction and the larger gas transport resistance at the interface. The diffusivity coefficient of CO_2_ decrease with an increase in the concentration of GO. This decrease in diffusivity coefficient is the indication of narrowing of the pore size of the MMMs, the decreased mobility of the interfacial polymer chains and increase in tortuosity [42] which generate the larger transport resistance for the gas with a larger molecular diameter and, therefore, higher selectivity for CO_2_/CH_4_ separation. The incorporation of the hybrid fillers thus significantly improve the selectivity due to the intensification of the diffusion process. At a higher concentration of GO increase in diffusivity and decrease in solubility is observed, which is assumed that the aggregation of the fillers reduces the active sites of the fillers for interaction with the CO_2._ It also might result in the formation of microvoids, resulting in higher permeance of gases but ultimately leading to a decrease in selectivity. 

**Figure 10:**
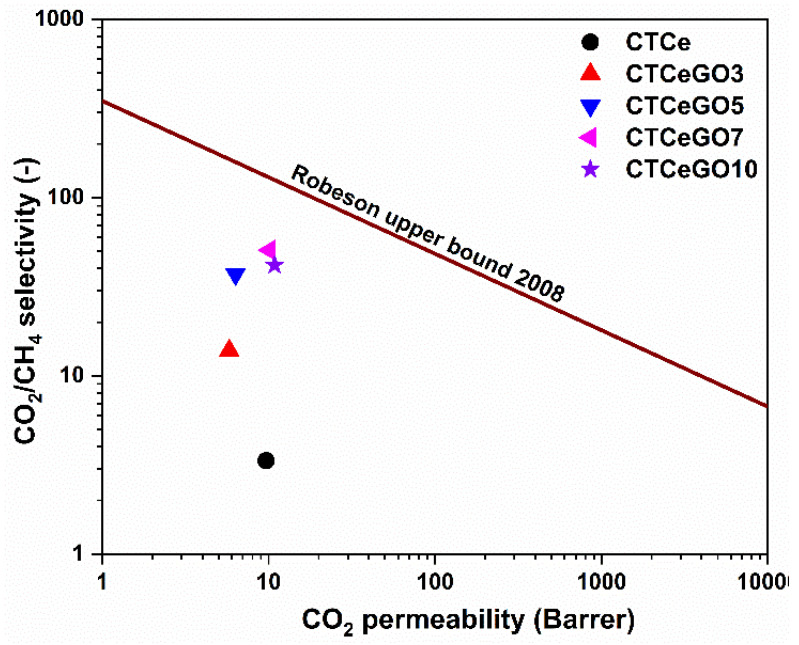
A comparison of CO_2_/CH_4_ gas separation efficiency of studied MMMs with Robeson 2008 upper-bound plot [4].

In order to compare the results with the literature data, the gas separation performance of the synthesised hybrid MMMs was compared with the Robeson upper bound plot, as shown in Figure 10. Although the gas separation performance cannot cross Robeson’s upper bound limit, the hybrid fillers’ use significantly increases the CO_2_/CH_4_ separation performance. Therefore, the simultaneous modification of the CeO_2_ filler with a small amount of GO leads to the detrimental improvement in the membrane efficiency exhibiting high potential value in the bio/natural gas separation field.

Furthermore, the gas separation performance of reported hybrid fillers based MMMs compared to those in this study is shown in Table 3. From this comparison, we deduce that blending the CeO_2_@GO fillers into the CTA matrix presented an attractive prospect for biogas/ natural gas upgrading. Therefore, we believe that the development of high-performance MMMs can be realised by optimizing the membrane preparation conditions and the gas separation parameters.

## 4. Conclusions

In summary, we have successfully synthesized hybrid fillers (CeO_2_@GO) incorporated CTA MMMs for the first time. The CTA-CeO_2_@GO MMMs were prepared by varying the GO concentration, and its effect on the gas separation performance was evaluated. An SEM-EDS image of the membrane cross-section shows uniform dispersion of the fillers in the polymer matrix. In contrast, the other physico-chemical analysis (XPS, FTIR, SEM and TEM) reveals the interfacial interaction between constituent components of the membrane. The fabricated hybrid MMMs demonstrated superior gas separation performance at an optimum GO loading concentration of 7 wt.% at 1.5 bar feed pressure reaching CO_2_/CH_4_ selectivity 50.7. This increment is almost 15 folds higher than the CTA-CeO_2_ MMMs and 1.5 fold higher than the CTA-GO membrane. The CO_2_/CH_4_ separation of the resulting MMMs was relatively near the upper bound region of the 2008 Robeson plotline. Thus, the gas permeation properties of the MMMs can be tailored efficiently even with the incorporation of extremely low loading of the fillers. Compared to other fillers requiring high filler loading to demonstrate enhancement of permeability/selectivity, using a low loading of fillers is advantageous given its possibility of mitigating particles aggregation and ensuring uniform distribution of the fillers in the polymer matrix. Thus, CTA membranes with a combination of two distinct nanofillers (CeO_2_@GO) have improved physico-chemical properties and thus show great potential for CO_2_ gas separation due to their synergistic effect. 

## Figures and Tables

**Table 1:** Mechanical properties of the synthesized membranes determined at 25 °C.

#	Stress (MPa)	Elongation at Maximum Stress (%)	Young’s Modulus (GPa)
CTCeGO3	36.3 ± 4.5	26.2 ± 4.5	1.02 ± 0.14
CTCeGO5	50.7 ± 3.3	18.9 ± 3.8	1.14 ± 0.15
CTCeGO7	99.5 ± 10.2	20.2 ± 6.1	1.31 ± 0.20
CTCeGO10	34.5 ± 7.1	19.5 ± 3.0	1.52 ± 0.11

**Table 2:** Permeability, selectivity and their corresponding solubility and diffusivity values of the synthesized MMMs.

#	Permeability (Barrer)		Selectivity (CO_2_/CH_4_)	Solubility Coefficient (10^−4^ mol m^−3^ Pa^−1^)		Diffusivity Coefficient (10^−12^ m^2^ s^−1^)	
	CO_2_	CH_4_		CO_2_	CH_4_	CO_2_	CH_4_
CTA-CeO_2_	9.67	2.89	3.35	1.33	0.35	1.6	5.4
CTCeGO3	5.81	0.42	13.83	31.71	0.89	0.61	1.57
CTCeGO5	6.33	0.17	37.23	42.99	0.01	0.49	1.70
CTCeGO7	10.14	0.20	50.7	79.66	0.72	0.44	1.20
CTCeGO10	10.87	0.26	41.81	14.85	0.47	2.29	1.42

**Table 3:** Gas separation performance of the reported MMMs in comparison with this study.

Membrane Type	Permeability (Barrer) /Selectivity (-)	References
Mixed porous fillers MOFs and zeolite silicate-1 blended with polysulfone [HUKUST-1/S1C-PSF (16 wt% filler mixture)]	P_CO2_ = 8.9 CO_2_/CH_4_ = 22.4	Zornoza et al. [11]
Ordered mesoporous silica and layered titanosilicate fillers with 6FDA-based copolyimide [MMMs (MCM-41(8Wt.%)+JDF-L1(4 wt.%) with 6FDA-4MPD/6FDA-DABA]	P_H2_ = 440 H_2_/CH_4_ = 32.0	Galve et al. [12]
GO and ZIF-8 blended with Polyethersulfone matrix followed by Pebax coating [2 rGO-ZIF-8-M]	P_CO2_ = N/A CO_2_/CH_4_ = 35.0	Jamil et al. [14]
MOF(UiO-66-NH_2_)@COF (TpPa-1) hybrid fillers in polysulfone matrix [5 wt% of MOF@COF fillers]	P_CO2_ = 7.1 CO_2_/CH_4_ = 46.7	Cheng et.al. [7]
CNT/SiO_2_ composite core incorporated into Pebax-1657 matrix	P_CO2_ = 148.3 CO_2_/N_2_ = 66.5	Wang et al. [5]
Ordered mesoporous silica(MCM-41) and MOF (NH_2_-MIL-53(AL) blended with polysulfones [8/8 wt.% of each MCM-41 and MOF]	P_H2_ =19.5 H_2_/CH_4_ = 67.3	Valero et.al. [13]
Cellulose-based poly-ionic liquid membranes P[CA][Tf2N]	P_CO2_ = 8.9 CO_2_/CH_4_ = 22.3	Nikolaeva et.al. [43]
Poly(butylene succinate)-cellulose triacetate blends [CTA + 10 wt% PBS]	P_CO2_ = 3.5 CO_2_/CH_4_ = 35.0	Cihal et al. [44]
PVA grafted on UiO-66-NH_2_ incorporated into polyvinyl amine matrix [24 wt% MOF]	P_CO2_ = 76.13 CO_2_/N_2_ = 45.6	Ashtiani et.al. [45]
CeO_2_@GO blended CTA membrane [7 wt.% GO with respect to CeO_2_ concentration]	P_CO2_ = 10.14 CO_2_/CH_4_ = 50.7	This work

## Data Availability

Not applicable.

## Supplementary Materials

The following are available online at https://www.mdpi.com/article/10.3390/membranes11100777/s1, Methodology for the synthesis of GO. Figure S1: EDS mapping of hybrid matrix confirming the presence of CeO_2_@GO. Figure S2: Surface roughness measurement of the synthesized membranes via 3D non-contact optical surface profiler; (A) CTCeGO3, (B) CTCeGO5, (C) CTCeGO7, and (D) CTCeGO10. Figure S3: EDS mapping of Ce throughout the cross-section in CTCeGO7 MMMs. Figure S4: XPS survey spectrum of CeO_2_@GO composite. Figure S5: FTIR spectra of a pristine GO. Figure S6: Permeability and their corresponding selectivity of different gases pair in CTCeGO7 MMMs. Table S1: Arithmetic mean height, root mean square height and a maximum height of the synthesized membranes determined by the 3D optical non-contact profiler. Table S2: Langmuir sorption isotherm fitting parameters for CO_2_ sorption in the CTA- CeO2@GO membrane matrix.
