# Supplementary material for: Improved CO2/CH4 Separation Properties of Cellulose Triacetate Mixed–Matrix Membranes with CeO2@GO Hybrid Fillers
Source: Membranes (Basel). 2021 Oct 11;11(10):777. doi: 10.3390/membranes11100777 (PMC8539915; doi:10.3390/membranes11100777)
Supplement: Supplementary file 1 [file membranes-11-00777-s001.zip › membranes-1404427-supplementary.pdf]

membranes

Supporting Information

Supplementary Materials: Improved CO_2/CH_4 Separation Properties of Cellulose Triacetate Mixed–Matrix Membranes with CeO_2@GO Hybrid Fillers

Chhabilal Regmi [1,*], Saeed Ashtiani [1], Zdeněk Sofer [2] and Karel Friess [1,*]

[1] Department of Physical Chemistry, University of Chemistry and Technology, Technická 5, 16628 Prague, Czech Republic; jamalias@vscht.cz
[2] Department of Inorganic Chemistry, University of Chemistry and Technology, Technická 5, 16628 Prague, Czech Republic; zdenek.sofer@vscht.cz
* Correspondence: regmic@vscht.cz; friessk@vscht.cz (C.R.); friessk@vscht.cz (K.F.)

Results

Methodology of GO synthesis

Synthesis of GO was based on the modified Hummers' method. The details of the synthesis process were explained elsewhere [1]. 10.32 g of graphite and 5.2 g sodium nitrate were mixed into 250 mL 1 M HCl with constant stirring. The temperature of the mixture was maintained to 0 °C by using an ice bath. 32 g of KMnO4 (Sigma-Aldrich, Czech Republic) was added to the mixture and continued stirring for 2 h. The temperature of the mixture was increased to room temperature and stirred for 4 h, followed by heating to 35 °C for 30 min. 250 mL of deionized (DI) water was added to the mixture and heated to 70 °C for 15 minutes. The mixture was then mixed with 1 L DI water. The excess MnO2/KMnO4 were removed by the addition of the 3% H2O2. GO was further washed by DI water and finally dried at 50 °C in a vacuum oven for 48 h.

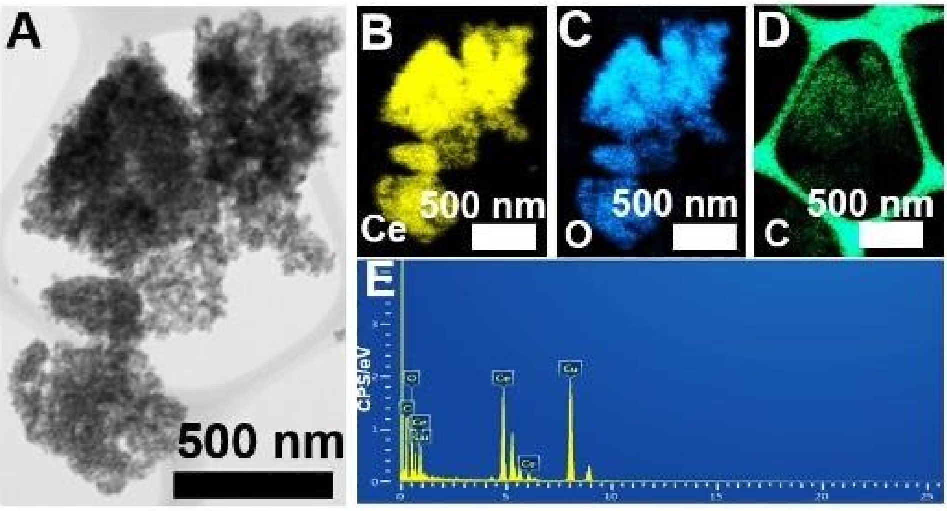

Figure S1. EDS mapping of hybrid matrix confirming the presence of CeO_2@GO.

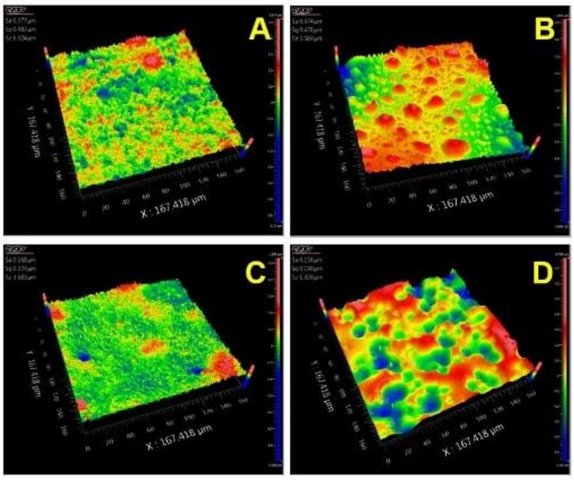

Figure S2. Surface roughness measurement of the synthesized membranes via 3D non-contact optical surface profiler; A) CTCeGO3, B) CTCeGO5, C) CTCeGO7, and D) CTCeGO10.

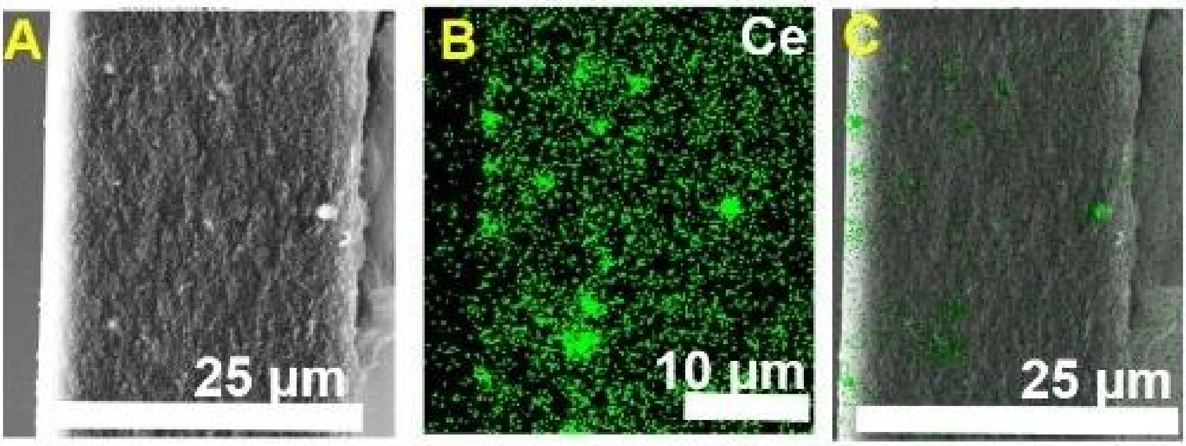

Figure S3. EDS mapping of Ce throughout the cross-section in CTCeGO7 MMMs.

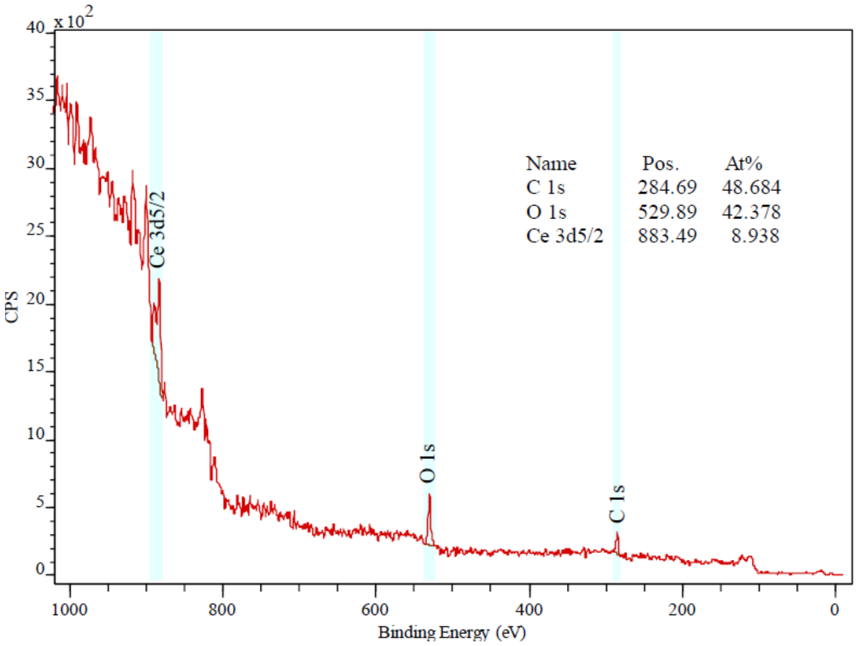

Figure S4. XPS survey spectrum of CeO_2@GO composite.

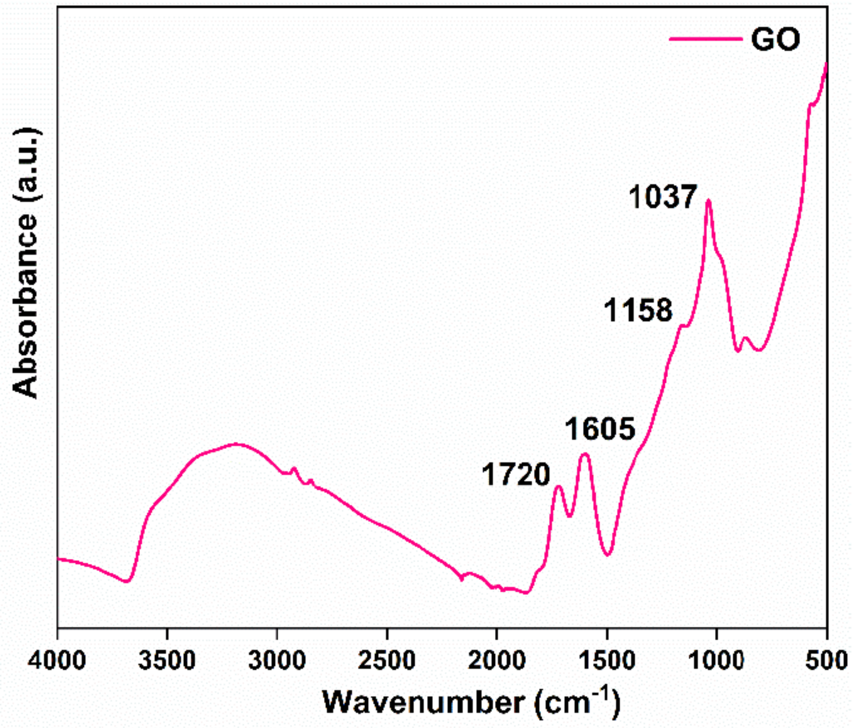

Figure S5. FTIR spectra of a pristine GO.

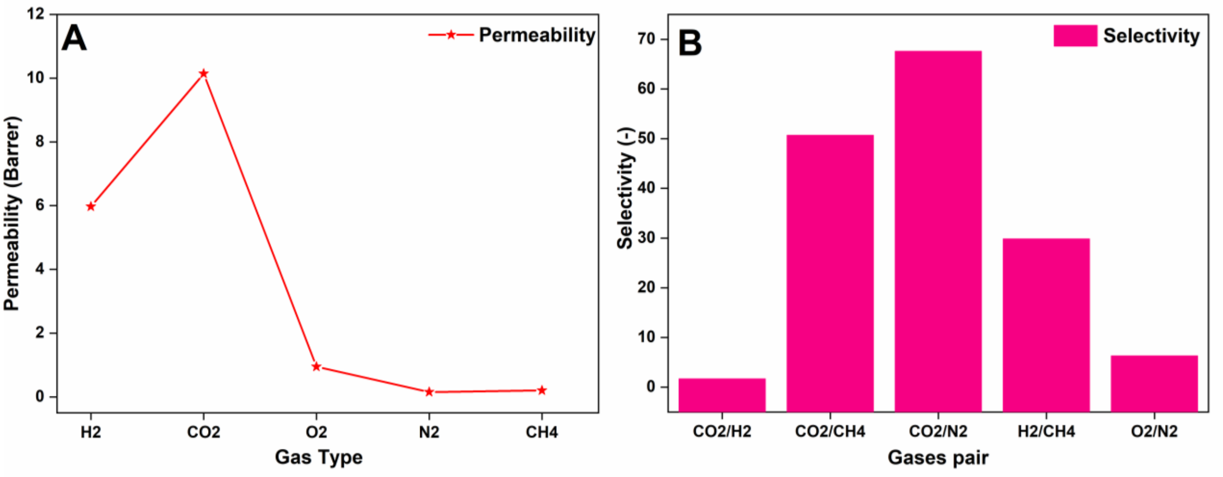

Figure S6. Permeability and their corresponding selectivity of different gases pair in CTCeGO7 MMMs.

Table S1. Arithmetic mean height, root mean square height and a maximum height of the synthesized membrane determined by the 3D optical non-contact profiler.

#	Arithmetic mean height (Sa)(μm)	Root mean square height (Sq) (μm)	Maximum height (Sz) (μm)
CTCeGO3	0.377	0.482	6.324
CTCeGO5	0.374	0.478	3.584
CTCeGO7	0.168	0.224	3.683
CTCeGO10	0.156	0.190	1.328

Table S2. Langmuir sorption isotherm fitting parameters for CO_2 sorption in the CTA- CeO_2@GO membrane matrix.

#	C_e	K_L	R^2
CTCeGO3	1132.28	0.03	0.997
CTCEGO5	141.96	0.39	0.997
CTCeGO7	122.78	0.53	0.997
CTCeGO10	97.59	0.91	0.996

References

1. S. Ashtiani, M. Khoshnamvand, A. Shaliutina-Kolešová, D. Bouša, Z. Sofer, K. Friess, Co0·5Ni0·5FeCrO4 spinel nanoparticles decorated with UiO-66-based metal-organic frameworks grafted onto GO and O-SWCNT for gas adsorption and water purification, Chemosphere, 255 (2020) 126966.
